# Measuring light scattering and absorption in corals with Inverse Spectroscopic Optical Coherence Tomography (ISOCT): a new tool for non-invasive monitoring

**DOI:** 10.1038/s41598-019-50658-3

**Published:** 2019-10-02

**Authors:** G. L. C. Spicer, A. Eid, D. Wangpraseurt, T. D. Swain, J. A. Winkelmann, J. Yi, M. Kühl, L. A. Marcelino, V. Backman

**Affiliations:** 1 Department of Chemical and Biological Engineering, Northwestern University, Evanston, IL USA; 2 Department of Biomedical Engineering, Northwestern University, Evanston, IL USA; 3 Department of Chemistry, University of Cambridge, Lensfield Road, UK; 4 Scripps Institution of Oceanography, University of California, San Diego, USA; 5 Department of Civil and Environmental Engineering, Northwestern University, Evanston, IL USA; 6 Integrative Research Center, Field Museum of Natural History, Chicago, IL USA; 7 Department of Medicine, Boston University School of Medicine, Boston, MA USA; 8 Marine Biological Section, Department of Biology, University of Copenhagen, Helsingør, Denmark; 9 Climate Change Cluster, University of Technology Sydney, Ultimo, NSW Australia; 10 Wellman Center for Photomedicine, Harvard Medical School and Massachusetts General Hospital, Boston, MA USA; 11 Department of Marine and Environmental Sciences, Nova Southeastern University, Dania Beach, FL USA

**Keywords:** Marine biology, Biomedical engineering

## Abstract

The success of reef-building corals for >200 million years has been dependent on the mutualistic interaction between the coral host and its photosynthetic endosymbiont dinoflagellates (family Symbiodiniaceae) that supply the coral host with nutrients and energy for growth and calcification. While multiple light scattering in coral tissue and skeleton significantly enhance the light microenvironment for Symbiodiniaceae, the mechanisms of light propagation in tissue and skeleton remain largely unknown due to a lack of technologies to measure the intrinsic optical properties of both compartments in live corals. Here we introduce ISOCT (inverse spectroscopic optical coherence tomography), a non-invasive approach to measure optical properties and three-dimensional morphology of living corals at micron- and nano-length scales, respectively, which are involved in the control of light propagation. ISOCT enables measurements of optical properties in the visible range and thus allows for characterization of the density of light harvesting pigments in coral. We used ISOCT to characterize the optical scattering coefficient (*μ*_*s*_) of the coral skeleton and chlorophyll *a* concentration of live coral tissue. ISOCT further characterized the overall micro- and nano-morphology of live tissue by measuring differences in the sub-micron spatial mass density distribution (*D*) that vary throughout the tissue and skeleton and give rise to light scattering, and this enabled estimates of the spatial directionality of light scattering, i.e., the anisotropy coefficient, *g*. Thus, ISOCT enables imaging of coral nanoscale structures and allows for quantifying light scattering and pigment absorption in live corals. ISOCT could thus be developed into an important tool for rapid, non-invasive monitoring of coral health, growth and photophysiology with unprecedented spatial resolution.

## Introduction

Reef-building corals provide critical habitat for as many as 1.3 million species^1^, which in turn directly support the lives of more than 500 million people^2^. The establishment, succession and maintenance of such coral reef ecosystems rely on a mutualistic endosymbiosis between a calcifying cnidarian animal and photosynthetic dinoflagellates of the family Symbiodiniaceae, which live within the coral tissue and provide the host with most of its energy demand through excretion of photosynthetically-fixed carbon^3^. Reef-building corals consist of thin tissue that deposits and covers a calcium carbonate skeleton, and such a configuration has evolved a variety of strategies to increase light availability to Symbiodiniaceae for greater photosynthate output, while minimizing light stress, which can typically occur in shallow waters especially when associated with high temperature^4,5^. Anomalously high temperatures and irradiances can impair photosynthetic efficiency leading to photodamage and production of reactive oxygen species (ROS), which may lead to the expulsion of the symbionts (coral bleaching) and result in death of the colony^5–9^. The trajectory of climate change is increasing the frequency and severity of coral bleaching events creating an urgent need to understand the mechanisms of light collection in corals^2,10,11^.

Different strategies to maximize light availability to Symbiodiniaceae have been described. Downwelling light that is not absorbed by the symbionts in the first pass can be scattered back into the tissue by the calcium carbonate skeleton and thus increase the light availability^12–17^. Within tissue, light scattering together with dynamic light redistribution due to tissue contraction and expansion can significantly increase light availability to symbionts^18–20^. Light-scattering or absorption by fluorescent host pigments can further modulate the light microenvironment of the Symbiodiniaceae, while also filtering out damaging short-wavelength radiation^8,21–23^ and affecting radiative heating of the coral tissue^24,25^. Corals can also actively regulate symbiont cell densities and thereby adjust microalgal self-shading within their tissues^26^.

Coral photophysiology is strongly affected by the light transport and absorption properties of coral tissue components and skeleton^18–20^. In coral tissue and skeleton, downwelling light undergoes both scattering and absorption, where spatial variations in refractive index lead to light scattering^19,27–29^. In tissue, refractive index variations are primarily due to intracellular structures, ranging from macromolecular complexes to organelles, and intercellular boundaries^30^. In the skeleton, light scattering is affected by variations in density of structures ranging from tens of nanometers in size (e.g., calcium carbonate nanograins, orientation and subsequent birefringence of the constitutive skeletal nanograins, organic matrix-skeleton boundaries) to micrometers (septa, corallites)^13,15,31^. The overall organization of these structures which are assembled hierarchically as a fractal^15,32,33^, can be characterized by their spatial autocorrelation function B_n_(r), with its functional shape parameterized by *D*, a fractal dimension^27,34^. Measuring *D* from the skeletons of 88 coral species revealed their overall skeletal organization to be a ‘mass-fractal’ structure (i.e., average *D* < 3), which correlated with their growth rates, light-scattering properties and bleaching susceptibility^31^. This emerging picture suggests that corals that grow at faster rates are more likely to have lower skeletal fractal-dimensions, denser skeletons, lower light-scattering properties, and higher bleaching susceptibility^13,15,31–33^. Coral tissue and skeleton differ in their respective absorption properties. Photosynthetic pigments within Symbiodiniaceae are the dominant light absorbers in tissue, where absorption spectra exhibit characteristic peaks corresponding to symbiont pigment composition^18,22,29,35,36^. Coral skeletons, on the other hand, are weakly absorbing with light transport dominated by scattering processes^13–15,37^.

Coral physiology and susceptibility to bleaching critically depend on light transport and absorption in tissues and skeleton. However, these processes are not fully understood, and many key questions remain open. For example, there is currently no consensus regarding whether light transport predominates in tissue or skeleton (e.g., due to a higher scattering coefficient) with opposing hypotheses being proposed^13,14,37^. In addition, it is not clear how spatially heterogeneous the scattering properties are across a coral. Mass fractal properties of coral skeletons are directly related to the physiology of skeletal deposition, as *D* of a coral skeleton is an end result of the balance between the rate of skeletal linear growth and the rate of mineral deposition, with both rates inherently linked with coral photophysiology and the chemical properties of the environment such as water acidity and temperature^31,38^. Understanding the mechanisms of light amplification in coral skeletons and tissue is crucial for coral photobiology and depends on the ability to model light transport, with either stochastic methods such as Monte Carlo, or deterministic solutions of Maxwell’s equations such as the pseudo-spectral time-domain (PSTD) method. Models, in turn, require an input of the accurate three-dimensional distributions of the scattering and absorption properties of coral skeletons and tissue, which are currently poorly understood due to the lack of techniques able to provide these measurements in live corals. Finally, the concentration of symbionts and pigments in coral tissue is critical in nearly every aspect of coral physiology, bleaching, and disease. Currently there is no non-destructive technique that is able to fully assess coral pigments in live corals. An ideal technology would allow non-invasive, real-time 3-D imaging of the key optical (absorption and scattering) and architectural (mass-fractal dimension *D*) properties in tissue and skeleton of live coral specimens.

Here we apply a variant of optical coherence tomography (OCT) to the study of coral optical properties. OCT is a non-invasive 3-D optical imaging technique that uses focused light typically in the near-infrared to probe and collect cross-sectional images from a sample that scatters light. First introduced in 1991, OCT has found widespread use in ophthalmology for clinical retinal imaging and a wide array of other biomedical applications including cardiology and dentistry^39,40^. OCT is a label-free technique that requires no sample preparation, relying on intrinsic differences in optical backscattering intensity within a sample to provide contrast. The transverse resolution is provided by scanning a focused beam across a sample and is determined by the numerical aperture of the focusing objective. The axial resolution is dependent on the center wavelength and bandwidth of illumination, and axial spatial resolution on the order of one micron can be achieved^41^. The depth of penetration depends on the scattering and absorption properties of the imaged object and in most biological tissues ranges from a few hundred microns to a millimeter. Recently, OCT has been used to image tissue and skeletons of live corals, providing complete 3-D structural images of tissue, skeleton, and chromatophore distribution not previously obtainable in a living coral^17^. In addition, the rapid imaging capability of OCT enabled live microscale study of coral polyp movement in response to high light illumination^17^.

While OCT holds a significant potential for coral imaging, its capabilities can be significantly enhanced to enable a comprehensive and quantitative imaging of coral optical properties. For a given resolution voxel, traditional OCT image intensity is proportional to the amount of backscattered light, making it impossible to decouple the effects of absorption and scattering, which would be required to measure pigment concentration and composition and the scattering properties of coral tissue^42^. Current techniques used to quantify pigment concentration in corals are all invasive and require the isolation of algal cells from the tissue, followed by pigment extraction with an organic solvent^43^. Pigment density is then normalized against an estimation of surface area from which the tissue was extracted. However, this approach is very time consuming and additionally prone to errors due to the architectural complexity of coral surfaces^44^. To quantify pigment density non-invasively, a complete characterization of coral optical properties is required. This involves the scattering coefficient, *μ*_*s*_, which quantifies the total amount of light scattered in all directions, the angular distribution of scattered light, *p*(*θ*), also known as the phase function, and the absorption coefficient, *μ*_*a*_. Another metric frequently employed in characterizing light transport properties of corals is the reduced scattering coefficient, ${\mu {\rm{^{\prime} }}}_{s}={\mu }_{s}(1-g)$, where the anisotropy factor (*g*) is equal to the average cosine of the phase function *p*(*θ*) and ${\mu {\rm{^{\prime} }}}_{s}$represents the inverse of the transport mean free path, the distance at which the trajectory of a photon in a medium becomes random.

Approaches to decouple these parameters included an OCT modality that, in addition to the backscattering coefficient, *μ*_*b*_, calculates the attenuation of the OCT signal in the longitudinal direction^45^. The latter is proportional to *μ*_*a*_ + *μ*_*s*_, integrated over the bandwidth and over several resolution voxels (typically ~50–100 μm). The phase function is then assumed to have a specific Henyey-Greenstein form, which was first developed to describe light scattering by interstellar dust and was later adopted to biomedical optics applications, and is parameterized by the single parameter *g*^46^. This links coefficients *μ*_*s*_ and *μ*_*b*_ and allows estimation of *μ*_*s*_ and *g*. This model assumes absorption is negligible, so it cannot be measured under this approximation. Furthermore, this functional form is only applicable in the very limiting case of a refractive index correlation function B_n_(r) with *D* = 3, which is not appropriate for coral light scattering since skeletal organization shows on average *D* < 3^15^.

Here we adopt a recently developed extension of OCT, inverse spectroscopic OCT (ISOCT), to obtain a comprehensive characterization of tissue and skeletal scattering, absorption, and architectural properties, which is independent of Henyey-Greenstein functional formalism and thus allows for evaluation of scattering properties rising from mass-fractal structures with *D* = <3 and *D* > 3. ISOCT measures the optical spectra of the OCT signal for any given 3-D voxel returning a 4-D data cube as a function of the voxel coordinates x, y, z, and wavelength λ^47–50^. In previous studies, ISOCT was used to measure *D* in mammalian tissues and cell culture models, showing that *D* is uniquely sensitive to the statistical structure of a medium in the length-scale domain from 30–450 nm^50^. These studies established the sensitivity of *D* to ultrastructural remodeling associated with biological processes such as condensation of chromatin in the cell nucleus, cytoskeletal remodeling, and crosslinking modification of the collagenous extracellular matrix, while assuming negligible optical absorption in these samples^47,49,51^. For the application of ISOCT on corals in the present study, the absorption spectra of prevalent pigments were measured *a priori*. We reasoned that because the narrow-band absorption spectra of pigments are drastically different from the broad-band, slowly varying spectra of backscattered light, *μ*_*s*_(λ), the pigment concentration and composition could be extracted from *μ*_*s*_(λ) + *μ*_*a*_(λ). Then *μ*_*s*_(λ) and *μ*_*a*_(λ) can be decoupled. Because the spectrum of *μ*_*b*_(λ) is functionally related to *p*(*θ*) through a Fourier transform, the combination of *μ*_*s*_ and *μ*_*b*_ can be used to extract *p*(*θ*) without making an approximation that *p*(*θ*) can be completely characterized by a single parameter $g$. In turn, the scattering properties can be used to estimate subdiffractional structural properties of the tissue and the skeleton, which provide further information on the growth pattern of a coral. In the present study, we use ISOCT to image the concentration and composition of tissue pigments, the scattering coefficient and the angular distribution of scattered light of both the tissue and the skeleton *in vivo*. These measures fully characterize light interaction and transport within a coral.

## Materials and Methods

### Inverse spectroscopic optical coherence tomography (ISOCT) instrument

The instrument used for ISOCT imaging has been described elsewhere^48,52^. For these studies, a visible (520–720 nm) spectral domain OCT system was used with a home-built spectrometer. The spectrometer camera frame rate was 45,000 frames per second, allowing for collection of a 512 × 512 pixel lateral scan in 5.8 seconds. Laser power at the sample did not exceed 10 mW during imaging, which in combination with the short scan time limited light exposure and potential phototoxicity to the coral. The visible spectrometer utilized a 4,096 pixel line-scan camera to obtain the spectrum of the OCT interferogram at each acquisition location across the sample. The spectral resolution of the spectrometer provided an axial imaging range of ~1.5 mm, allowing for sufficient imaging depth in the spatial domain to image through weakly scattering coral tissue in the visible band. A detailed description of methods for OCT spatial resolution measurement is included in Supplementary Material. The lateral resolution was calculated to be 8.8 μm, with full imaging axial resolution measured to be 1.22 μm in air (Supplementary Fig. 1B). For the spectrally-resolved OCT imaging required in ISOCT signal analysis, the axial imaging resolution was measured to be 7.88 μm, as previously reported^52^.

### Live coral imaging

Live specimens of the reef-building corals *Diploastrea heliopora* and *Merulina ampliata* were obtained from the aquarium trade and from the Shedd Aquarium, Chicago, IL who approved the coral species used in this study for research by their institutional review board, i.e., no endangered or threatened species as listed by the US Endangered Species was used in this study. Corals were kept in a saltwater tank (JBJ Nano Cube 28 gallon) with two Acela pumps rated at 1000 L per hour and a 105 W quad compact fluorescent lamp (10,000 K, 3.75 W/gallon), maintained at a temperature of 26.5 ± 0.5 °C. ISOCT images were acquired by placing the coral in a shallow dish, removing the majority of water covering the coral tissue and immediately scanning the focused sample laser beam over the region of interest, a process that lasted less than 10 seconds.

To determine the location of the skeleton in the OCT images, a preliminary scan of a selected region of coenosarc tissue was acquired. Then, while covering the OCT objective, a Waterpik system was used to wash away all of the coral tissue within the previously scanned region. Care was taken to prevent the coral frag from moving during this process, and a follow-up scan of the same region with the same instrument settings was acquired. For both scans, water covering the sample was kept to a minimal depth ~1 mm to ensure accurate vertical colocalization between the scans before and after tissue removal.

### Light scattering model for ISOCT signal analysis

Light propagation through a turbid medium, including a biological tissue, is fully determined by the spatial distribution of the complex optical refractive index n(x, y, z, λ)^27,53^. Spatial variations in the absolute value of refractive index give rise to light scattering, while the imaginary part gives rise to light absorption. The respective fundamental optical properties are the differential scattering cross section function σ_s_(θ, λ, x, y, x) [cm^2^], which describes the amplitude of light scattering as a function of scattering angle θ, and the absorption coefficient *μ*_*a*_(x, y, z, λ) [cm^−1^], described as the probability of light absorption per infinitesimal path length. Under the first-order Born approximation, which generally describes a medium with relatively weak scattering potential and is broadly applicable to most biological tissues, σ_s_ is related to the autocorrelation function of n(x, y, z) via a Fourier transform^54^. For a given (x, y, z) position within a turbid medium, scattering properties are described via the scattering coefficient, *μ*_*s*_ [cm^−1^], which is the integral of σ_s_ per unit volume over all directions, and the angular (phase) function of scattering *p(θ)*^55^. Other properties, such as the backscattering coefficient *µ*_*b*_, which describes the likelihood of an incident photon scattering in the direction of origin, can also be derived from σ_s_. The ISOCT signal is scaled by *µ*_*b*_, and in order to relate the ISOCT signal to the optical properties of the medium, we model the autocorrelation of the refractive index distribution *B*_*n*_ using the three-parameter Whittle-Matérn model,

1 $${B}_{n}(r)={A}_{n}{(\frac{r}{{L}_{n}})}^{(D-3)/2}{K}_{(D-3)/2}(\frac{r}{{L}_{n}})$$

where *r* is the spatial separation between any two points, *A*_*n*_ is proportional to the variance of the refractive index *σ*_*n*_, *L*_*n*_ represents a correlation distance, *D* describes the shape of the correlation function, and *K*_*x*_*()* denotes the modified Bessel function of the second kind^27^. Because the exact form of *B*_*n*_ cannot be known *a priori* and may vary not only across different types of biological tissue but even within the same tissue type, an advantage of this model is its versatility. Parameterized by *D*, the majority of the realistic functional forms of the autocorrelation function are well represented by the model and can be ascertained experimentally, thus eliminating the need to assume a specific form *a priori*. For *D* < 3, the autocorrelation function has an inverse power-law form typical of a fractal tissue organization with the fractal dimension *D*. For 3 < *D* < 4, the autocorrelation function is a stretched exponential function. It is an exponential function with attenuation length *L*_*n*_ for *D* = 4 and a Gaussian for *D* approaching infinity. Physically, *D* is a fractal dimension, characterizing the degree of self-similarity of structures at different length scales, and can alternatively be thought to characterize the degree of clumpiness in a continuous random medium. Enhanced backscattering spectroscopy measurement of *D* from various hard and soft scattering structures is reported in Figure 6 from a prior publication^15^, with mean *D* values from samples ranging from ~2 for to >3. Earlier techniques attempting to measure *μ*_*s*_ and *g* frequently relied on the approximation of *p*(*θ*) as the Henyey-Greenstein function, which corresponds to a special case of the Whittle-Matérn model for *D* = 3^14,37,55,56^. However, the shape of *p*(*θ*) varies considerably with changes in *D*, as can be appreciated from Supplementary Fig. 2(A) which shows an example of *p*(*θ*) behavior for *D* ranging from 1 to 5. Scattering behavior is also strongly influenced by changes in both *D* and *g*, as can be observed in Supplementary Fig. 2(B) which shows the behavior of *μ*_*s*_ as a function of *D* and *g*.

Applied to the imaging of coral tissue, ISOCT measures the optical backscattering intensity, *I*_*b*_(λ, x, y, z), the square of which is proportional to the product of *μ*_*b*_(λ, x, y, z) of the sample and the attenuation term integrated over the depth above the voxel of interest, which depends on the sum of the scattering and absorption coefficients of the medium, *μ*_*t*_ = *μ*_*s*_(λ) + *μ*_*a*_(λ), for a given voxel. These relative contributions of scattering and absorption were fit using a MatLab code implementing the Nelder-Mead algorithm^57^ to iteratively minimize the sum of square differences as mathematically described by the following cost function:

2 $$cost({c}_{a},\,{c}_{s},\,D,\,{L}_{n})=\,\mathop{\sum }\limits_{{\lambda }_{i=1}}^{{\lambda }_{i=N}}\,{|{\mu }_{t}^{\ast }({\lambda }_{i})-f({c}_{a},{c}_{s},D,{L}_{n},{\lambda }_{i})|}^{2}$$

where $f({c}_{a},\,\,{c}_{s},\,D,\,{L}_{n})=\,{c}_{a}\,{\mu }_{a}^{\ast }({\lambda }_{i})+{c}_{s}\,{\mu }_{s}^{\ast }({\lambda }_{i},\,D,\,{L}_{n})$; $\,{c}_{a},\,{c}_{s}\,\in [0,\,\frac{1}{2}]$. For simplicity, we introduce * to denote where a metric was normalized by its maximum along wavelength. Thus, the absolute coefficients are defined as:

3 $${\mu }_{a}(\lambda )={c}_{a}\,{\mu }_{a}^{\ast }(\lambda )\,{\rm{\max }}\,\{{\mu }_{t}({\lambda }_{i})\}$$

4 $${\mu }_{s}(\lambda )={c}_{s}\,{\mu }_{s}^{\ast }(\lambda ,\,D,\,{L}_{n})max\,\{{\mu }_{t}({\lambda }_{i})\}$$

Using Eq. (2), the effect of absorption is estimated from the attenuation coefficient spectrum based on the characteristic absorption spectrum that is distinct from the scattering spectrum, which decouples *μ*_*s*_(λ) and *μ*_*a*_(λ). (see ‘ISOCT analysis’ section in Results for details on the estimated absorption spectra). The next step is to determine *D* within a given resolution voxel (approximately 9 × 9 × 1.2 μm) of the sample by fitting *µ*_*b*_(λ) using the Whittle-Matérn model: *µ*_*b*_(λ)$\,\propto \,{\lambda }^{\frac{D}{2}-2}$^48^. As a result, ISOCT measures the set of three parameters (optical parameters *μ*_*s*_ and *μ*_*b*_ and the ultrastructural parameter *D*) in 3D. The Whittle-Matérn model is then used to calculate *g* and ${\mu }_{s}\text{'}$ using equation (6) from a previous publication^27^. Furthermore, one can fully characterize all three ultrastructural parameters (*A*_*n*_, *L*_*n*_, *D* from Eq. 1) from the measured optical properties (*μ*_*s*_, *μ*_*b*_) for a more thorough characterization of the structure of the medium^27^. The analysis of ISOCT in coral skeletons is performed with the same algorithm; however, in coral skeletons, as opposed to coral tissue, *μ*_*s*_(λ) ≫ *μ*_*a*_(λ)^15^ and skeletal light absorption is ignored.

### ISOCT signal analysis

**Figure 1:**
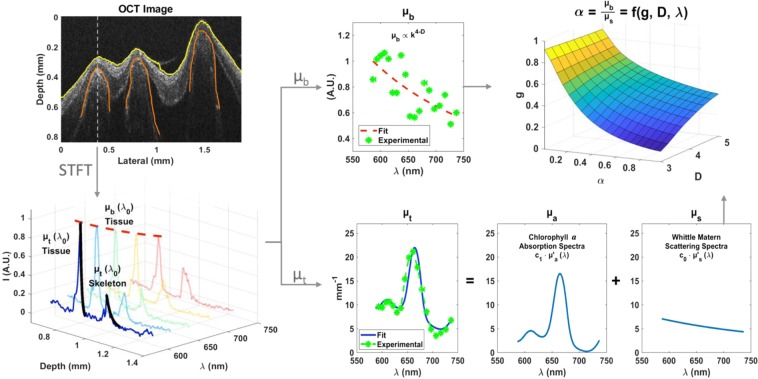
Flowchart for ISOCT data processing. Processing starts with the acquisition of the OCT volume and detection of surfaces of tissue and skeleton. From a short-time Fourier transform, the spectral cube is generated and wavelength-dependent backscattering and attenuation coefficients are computed. Fractal dimension *D* is fit from *μ*_*b*_, and the *μ*_*s*_ spectral shape and scaling factors for *μ*_*a*_ and *μ*_*s*_ are also fit from the spectrum of *μ*_*t*_. Finally, the wavelength-dependent (*g*) is computed from the albedo (*α*) and *D*.

A full description of the steps for processing ISOCT data is given in Results, along with a processing flowchart in Fig. 1. Briefly, the spectral domain OCT data collected at wavelengths between 520 and 720 nm were converted to spectrally-dependent OCT images through a short-time Fourier transform (STFT) with 22 separate Gaussian windows, each with full width at half-maximum (FWHM) of 20 nm. System spectral response was accounted for by calibration with scans of a reference solution of 80 nm polystyrene beads in water, approximating a medium with Rayleigh scattering behavior. Next, surfaces of either coral tissue or skeleton were detected with either an edge detection algorithm or manual segmentation. The surface edge detection algorithm was implemented through a custom MatLab script and operated on each OCT B-scan in the image cube by first filtering and adjusting intensity values to optimize image contrast. Then, an extended-maxima transform was applied to binarize the image, delineating the B-scan region containing scattering sample. Binary morphological operations then detected the top surface of this sample region, which when combined with all B-scans provided a 2-D sample surface profile image for the 3-D OCT scan. The signal intensity decay as a function of depth below the detected edge was fit with an exponential function over a 90 μm depth to quantify the total attenuation coefficient, *μ*_*t*_, for each OCT A-line for each wavelength. This method for measuring *μ*_*t*_ was verified by applying it to samples of polystyrene spheres with known magnitude of *μ*_*t*_ across a *μ*_*t*_ range relevant for tissue (see details of validation analysis in Supplementary Fig. 3 and Supplementary Material). The full spectrum of *μ*_*t*_ was then decomposed into a linear combination of scattering and absorption coefficients assuming an absorption dominated by chlorophyll *a* (see ISOCT analysis section in results regarding how the shape of *μ*_*t*_ spectra showed a characteristic peak of chlorophyll *a* absorption, which was the predominant peak for the corals measured). The true backscattering spectrum from each voxel was measured by dividing the OCT spectrum by the spectrally-dependent *μ*_*t*_ attenuation term above the voxel. This corrected spectrum was then fit to the power-law relation with wavelength shown above to determine *D*.

For calculation of chlorophyll *a* density, the linearly decomposed spectrum of *μ*_*t*_, consisting of the respective fractional contributions of *μ*_*s*_ and *μ*_*a*_ was taken with the full spectral shape of *μ*_*a*_ assumed to be that of chlorophyll *a* in methanol. This absorption spectrum was taken from values collected by J. Li and provided on the Oregon Medical Laser Center webpage^58^, and shows a characteristic peak in the visible around 660 nm. The spectroscopic resampling of OCT data was performed with STFT window FWHM of 20 nm, meaning that absorption peaks from other pigments that fall outside this width from the peak at 660 nm would have negligible contribution to the linear regression used to compute *μ*_*a*_ contribution and would not contribute to the calculation of chlorophyll *a* concentration. Chlorophyll *a* concentration was then calculated by dividing the measured absorption coefficient by the molar extinction coefficient provided, scaled by the peak intensity at 660 nm. This molar concentration was converted to areal density [mg cm^−2^] by molar conversion of chlorophyll *a* and integrating the volumetric concentration over the 90 μm thickness of tissue used for *μ*_*t*_ measurement.

## Results

### ISOCT analysis

Here we describe a novel OCT data processing method employed to extract structural and optical properties from OCT data (Fig. 1). A short-time Fourier Transform was applied to the raw OCT data using Gaussian windows (FWHM of 20 nm) to compute a spectral cube consisting of OCT intensity as a function of 3-D position and wavelength. This data cube is collected by raster scanning the OCT sample laser beam across the sample surface, collecting an OCT A-line, or the backscatter intensity function versus depth, from each transverse position. The series of A-lines from a single scan across the sample form an OCT B-scan image, which displays a depth-slice of backscattering intensity from the sample in question. From OCT B-scan images the surface of the coral tissue and skeleton were detected, and the wavelength dependent attenuation coefficient, *μ*_*t*_, was computed from the spectral cube by fitting an exponential fit in depth at a fixed thickness of 90 μm below each surface for every lateral position according to the Beer-Lambert law. This thickness was selected in order to provide sufficient depth information within the constraints of axial resolution of the spectrally resolved OCT images and optical penetration through the coral tissue and skeleton under study. This technique for calculating *μ*_*t*_ was validated for by measuring standard aqueous suspensions of 200 nm polystyrene spheres with known *μ*_*t*_ from Mie theory (see Supporting Methods and Supplementary Fig. 3). The backscattering coefficient *μ*_*b*_ was calculated for the tissue by averaging signal intensity from the first 90 μm of tissue from the surface. Using the thickness of the tissue layer and calculated attenuation coefficient, the decaying signal intensity in the cube was compensated by the spectrally dependent attenuation in order to yield an accurate backscattering spectral shape. From this modified spectral cube, the backscattering coefficient of the skeleton was computed by averaging the upper 90 μm of the skeletal layer.

Next, from the backscattering coefficient of the tissue and skeleton, the first-order Born approximation was employed, reducing the spectral dependence to a simple power law fit with fractal dimension *D* (Fig. 1C). *D* was fit for every lateral position of the tissue and skeleton where the surface edge was available from an extended-maxima transform based algorithm for the tissue surface or manual segmentation for the skeleton surface, specular reflection was absent, and topography was within the roll-off sensitivity range of the OCT instrument, which is due primarily to spectral resolution of the spectrometer and is on the order of −10 dB mm^−1^ in air for the visible spectrometer used in this study.

From *μ*_*t*_*(λ)* of the tissue layer and the skeleton layer, the *μ*_*s*_ spectral shape was fit by assuming that the Whittle-Matérn functional form can parameterize the statistics of the local refractive index variations using three structurally relevant parameters, *σ*_*n*_, *D*, and *L*_*n*_^27^, see methods. Additionally, we observed that the shape of *μ*_*t*_ spectra showed a characteristic peak of chlorophyll *a* absorption. For the corals measured, this was the predominant absorption feature observed and so for this model, the predominant absorbing pigment of chlorophyll *a* was assumed to be responsible for all absorption within the coral tissue. Using this assumption, the relative contributions of *μ*_*a*_ and *μ*_*s*_ to the total *μ*_*t*_ spectrum were fit using the Nelder-Mead algorithm described in “Light scattering model for ISOCT signal analysis” in Materials and methods in order to minimize the cost function of deviation from the experimentally measured total attenuation spectrum. To scale measured *μ*_*b*_ intensity, a standard sample of 80 nm polystyrene sphere suspension with known scattering properties was measured for calibration. Finally, the ratio of *μ*_*b*_ to *μ*_*s*_, known as the reflection albedo *α*, is related to the structural parameters *D* and *g* within the Born approximation and is described in a prior publication^27^. Using this relationship, a lookup table was computed to determine *g* for given *α* and *D*.

### Mapping structural and optical properties and chlorophyll *a* surface density measurement

**Figure 2:**
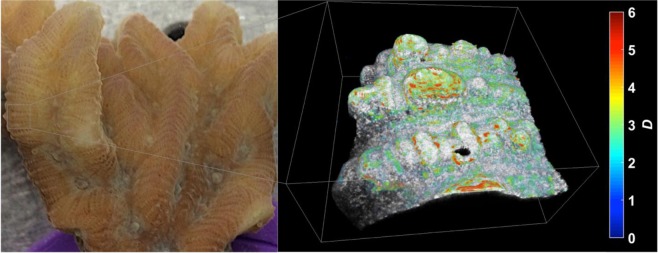
Example rendering of ISOCT data from *Merulina ampliata*. Photograph of live coral measured shown on left, with box demarcating region scanned. 3-D volumetric rendering of OCT scan volume shown on right, with traditional OCT image intensity shown in grayscale with 3-D overlay of local *D* value in color. Rendering box is 2.56 × 2.56 × 1.5 mm.

To measure structural and optical properties from the tissue and skeleton of live corals, we scanned specific regions on specimen fragments of *Merulina ampliata* and *Diploastrea heliopora* with our visible OCT system. The 3-D nature and micron-scale resolution of visible OCT imaging can be appreciated in Supplementary Video 1, which shows a flythrough of OCT B-scans of a *Diploastrea heliopora* polyp mouth. The spectrally enabled ability of ISOCT to quantify tissue structure through its fractal dimension *D* is also demonstrated in Fig. 2 (see Supplementary Video 2 for a rendered scan volume of the coenosarc region of *Merulina ampliata*). In this rendering, the traditional OCT intensity is depicted as a grayscale image and *D* values are represented by color hue. It is apparent that in coral tissue, *D* has a heterogeneous spatial distribution with certain tissue regions showing *D* as low as 1.5 while others show *D* values as high as 5. Furthermore, it is seen that for many regions in the tissue, *D* does not reflect a value sufficiently close to 3 to make the assumption of a Henyey-Greenstein scattering phase function appropriate.

**Figure 3:**
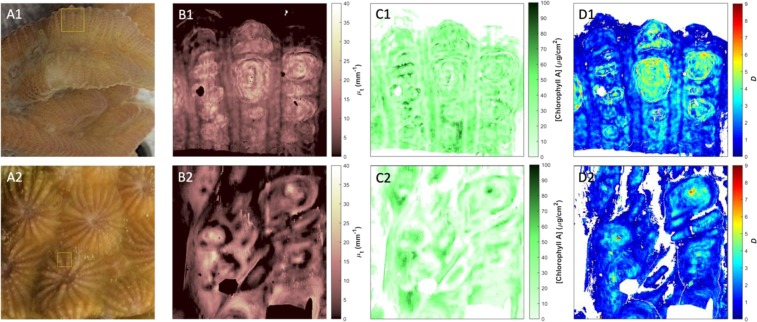
Top-down projection maps of optical and structural properties. Photographs of *Merulina ampliata* (**A1**) and *Diploastrea heliopora* (**A2**) with box demarcating region scanned with ISOCT. *En face* projection maps of total attenuation coefficient *μ*_*t*_ (**B1**,**B2**), chlorophyll *a* concentration (**C1**,**C2**), and *D* value (**D1**,**D2**) for *Merulina* and *Diploastrea*, respectively. Maps (**B1**,**C1**,**D1**) are 2.56 × 2.56 mm; maps (**B2**,**C2**,**D2**) are 1.85 × 1.85 mm.

Linking the ultrastructural characteristics and corresponding optical properties of corals is fundamental for mechanistic understanding of light transport in these ecologically important organisms. ISOCT provides an unparalleled method for mapping the spatial distribution of these properties with high spatial resolution. Maps of some of these properties, namely the total attenuation coefficient *μ*_*t*_, areal density of chlorophyll *a*, which is directly related to *μ*_*a*_, and *D* from representative regions of coenosarc tissue in *Merulina ampliata* and *Diploastrea heliopora* are shown in Fig. 3. An example projection map of scattering anisotropy, *g*, calculated from the measured scattering albedo and *D*, is shown in Supplementary Fig. 4. Accompanying histograms of calculated *g* distributions from tissue and skeleton of *Merulina ampliata* and *Diploastrea heliopora* show that both compartments have *g* close to 1 indicating high forward scattering from these structures.

While the penetration of visible OCT is somewhat limited through the highly absorbing and scattering tissues of many corals, variability in tissue thickness and skeletal topography allows for imaging directly through living tissue and quantifying optical and structural properties from certain areas of the coral coenosteum, i.e., the skeletal material formed below the connective tissue between polyps. To confirm the location of the skeleton in these regions, we captured OCT scans from a specific region of coenosarc in *Diploastrea heliopora* before and after tissue removal (see peaks of skeletal plates easily resolved through tissue in Supplementary Video 3). Maps of total attenuation coefficient *μ*_*t*_, chlorophyll *a* concentration, and *D* of *Merulina ampliata* and *Diploastrea heliopora* skeletons are shown in Supplementary Fig. 5. Chlorophyll *a* areal density was determined by ISOCT for the first 90 µm of tissue thickness for surface areas over skeletal ridges and valleys (Fig. 3C1 and C2, respectively, see inset in Fig. 3A1 and A2). While considerable spatial variability was observed across skeletal ridges and valleys (from 0.05–0.1 mg cm^−2^), the average chlorophyll *a* density was 29.5 ± 14.4 µg cm^−2^ for *Merulina ampliata* and 17.9 ± 15.8 µg cm^−2^ for *Diploastrea heliopora*.

**Figure 4:**
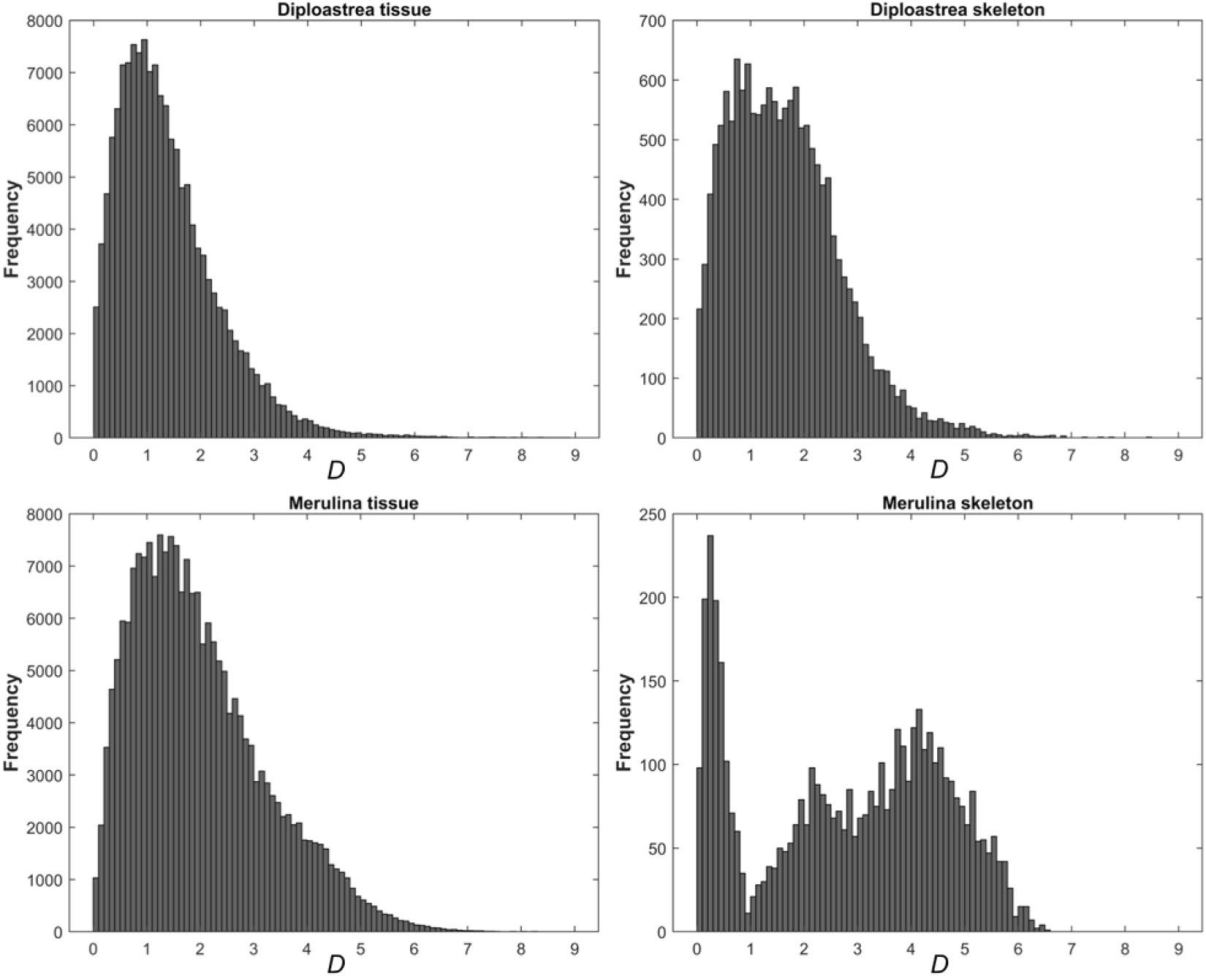
Histograms of *D* values from tissue and skeletal compartments of *Merulina ampliata* and *Diploastrea heliopora*. Frequency plotted reflects likelihood of a 5 × 5 μm pixel having a given *D* value.

Spatial variability of all three of these properties in the projected view is apparent and of consequence when modeling light transport within the whole colony. A large variability in the structural parameter *D* was observed for the tissue and skeletal compartments of *Merulina ampliata* and *Diploastrea heliopora* (Fig. 4). Measured spatial variance can be considered as the sum of true spatial variance and instrumental variance. Instrument variance can be assessed when measuring the spatial variability of a truly homogenous sample. To determine instrument variance, we measured an aqueous suspension of 80 nm polystyrene beads at a concentration of 2 wt%, a medium reasonably homogenous considering each OCT resolution voxel contains on the order of 10^4^ particles. We applied the same ISOCT analysis algorithm to a scan of these beads as was applied to the data acquired from coral tissues, and used a randomized square averaging box to randomly sample *D* values at various locations on a projected *D* map. By plotting the standard deviation of *D* values from this random sampling with respect to the edge length of the averaging box, the degree of spatial variance is expressed (Supplementary Fig. 6). Results from an example scan of *Merulina* tissue thus yielded a significantly higher spatial variance than the intrinsic variance of the ISOCT instrument.

## Discussion

In this proof-of-concept study, we demonstrate that visible light ISOCT, which combines the structural and chemical analytic capabilities of spectroscopy with the high-resolution 3-D imaging of OCT, enables the non-invasive mapping of fundamental optical properties of coral tissue including chlorophyll *a* concentration, the absorption coefficient, the scattering and the reduced scattering coefficients, and the critical parameters governing the angular distribution of scattered light (*g* and *D*). In addition, ultrastructural properties such as the variance of the refractive index within tissue and the correlation length scale of the refractive index variations can also be estimated. This ability to non-invasively extract and 3-D map fundamental optical and structural properties in living corals is a breakthrough in coral biology, which now allows the quantification of optical parameters needed for rigorous simulations of light transport in corals.

The spectral decomposition in ISOCT enables more accurate and more complete characterization of light scattering in coral tissue. Previous studies of optical transport in corals have focused on coral controls of Symbiodiniaceae light exposure due to tissue scattering and lateral light transport^19,37^ and skeletal scattering^13,15,59^. Hitherto, simulations of light propagation in corals have largely relied on the assumption that light scattering direction in coral tissue is dictated by the Henyey-Greenstein phase function, which is a special case of the Whittle-Matérn phase function when *D* = 3^37,50^. ISOCT spectral decomposition now enables the actual measurement of *D* in living coral tissue, thus removing the need for the Henyey-Greenstein approximation. Indeed, as seen in Fig. 2, coral tissue *D* values vary significantly with location. This variability is an inherent property of coral tissue and is significantly greater than the technical variability of measuring *D*. By precisely measuring *D* and *g*, accurate information regarding the scattering phase function can now be implemented in more refined models of light propagation in the coral tissue. With the capability of ISOCT to measure spatially resolved ultrastructure and optical properties, we foresee this technique to significantly enhance current understanding of light propagation throughout the coral holobiont. Beyond photophysiology, *D* is a key descriptor of coral tissue and skeletal ultrastructure and growth pattern, which can be used e.g. as a proxy for the rate of coral calcification^15,31^, and in tissue, *D* of cell nuclei has been shown to be a critical regulator of transcriptional plasticity^60^.

There are, however, limitations of ISOCT-based measurements of optical and structural properties that must be considered for corals. First, the limited penetration depth of OCT imaging in the visible spectral range is restricted to superficial regions of tissue and skeleton. In fact, where coral tissue was thick in regions between skeletal plates or protrusions, the coral skeleton was not visible with our OCT system. The difficulty in imaging through thick coral tissue is compounded by the limitations in effective working range, as all Fourier domain OCT systems have a roll-off in image intensity with depth^61^. While the limitation of roll-off can be somewhat alleviated through motorized stage control and application of automated surface detection and stitching algorithms, the limited optical penetration in the visible range required for analysis of chlorophyll *a* concentration restricts application of this technique to the soft tissue compartment and superficial skeleton only in regions where tissue is thin. Additionally, the analysis method presented here relies on several assumptions regarding the coral tissue structure in order to determine the optical and structural properties presented. In the calculation of *μ*_*t*_ from each compartment, it was thus assumed that the medium was randomly homogeneous in keeping with Beer-Lambert law along the 90 μm depth of fitting. While this is a fair assumption for many tissue regions, the region of averaging, in some cases, might include distinct layers of epidermis, mesoglea, and gastrodermis, which may have different attenuation properties. For a coarse modeling of optical transport, such bulk properties may still be of use, but a strength of OCT in this application is its measurement of optical properties with high spatial resolution that will allow for a more detailed analysis of photon propagation in these specific tissue compartments in future study. For instance, transmission electron microscopy (TEM) has been used to image the nanoscale structure of tissue as an independent metric of the statistical mass-density autocorrelation function characterized with ISOCT^62,63^. These complementary techniques can be applied to each compartment of the tissue to fully characterize the structural properties and scattering therein. The coral skeleton presents a separate challenge with topographic roughness, and optical properties in this study were only measured from the top of septal plates where structure within the skeleton appeared homogeneous in OCT B-scans. While the interpretation of anatomical features in OCT scans of living corals remains unclear for certain species, the tissue stripping presented in Supplementary Video 3 confirms septal plates are clearly discernable through the tissue with visible OCT.

The linear fitting used to separate scattering and absorption coefficients from *μ*_*t*_ relied on the assumption that chlorophyll *a* was the predominant absorber present in the coral tissue, simplifying this fitting procedure. A more detailed analysis taking into account additional absorbing pigments in the coral tissue such as endogenous fluorophores, fluorescent and non-fluorescent host pigments, in pigment granules^22,24^ is feasible, provided the pigment absorption spectrum is known *a priori* and it contains a distinctive feature within the visible wavelength band analyzed. Fitting the measured backscattering spectrum to calculate fractal dimension, *D*, relies on the assumption that the autocorrelation function of the medium can be modeled as a Whittle-Matérn function and that the first-order Born approximation applies. These approximations have been applied to the analysis of mammalian tissues previously^27,28,48,50^, and when the influence of absorbing pigments is properly compensated should also be applicable to media such as coral tissue. The average chlorophyll *a* areal density mapped in Fig. 3 is 29.5 ± 14.4 µg cm^−2^ for *Merulina ampliata* and 17.9 ± 15.8 µg cm^−2^ for *Diploastrea heliopora*. These values are comparable to chlorophyll *a* concentrations measured in tissues of several species of healthy corals, which can range between 3 and 50 µg cm^−2^ ^64–69^.

ISOCT allows for direct measurement of optical properties and 3-D micro-morphology in living corals, which will facilitate the understanding of light propagation and radiative energy budgets in corals. This is key to a better mechanistic understanding of coral photophysiology and thermal resilience in a changing climate. The emerging picture is that of a tissue-skeletal system that modulates optical and thermal properties of corals through a variety of strategies to optimize light regimes and heating for the photosynthetic algal symbionts harbored in their tissues. The strategies uncovered range from skeletal- and tissue- light scattering that increase symbiont light exposure^13,15,16,18,29,37,70^ to fluorescent pigments in the tissue that modulate light absorption, light scattering and heating in the tissue^22,24,25,71^ to dynamic light redistribution resulting from tissue contraction and expansion^18–20^. Skeletal light scattering has been implicated in the acceleration of light stress of *in hospite* Symbiodiniaceae under increased temperature by a positive feedback-loop between photodamage and symbiont loss that results in coral bleaching^13,15,16,70^ and can affect coral recovery from bleaching events^72^. Green fluorescent protein-like host pigments have been shown to reduce coral tissue heating and light absorption by increased light scattering at high concentrations of pigment, which enhances reflectivity and thus diminishes light penetration further into the tissue leading to reduced tissue heating^24^. These different properties vary significantly among coral species resulting in unique light microenvironments for the symbiont algae and distinct stress-mitigating strategies^15,16,18,19,73^. Imaging both skeletal and tissue compartments in intact living corals will help to further elucidate different photophysiology responses to thermal stress, which is an exciting avenue of ongoing coral research utilizing ISOCT. Current developments in low cost and portable OCT technology could further enable the application of this ISOCT approach to study live corals in their native habitat^74,75^.

## Conclusion

Here we introduce the novel application of ISOCT to study the microscale structure and optical properties of live corals. We believe this technique to be unique for this application in its ease of use, imaging speed, spectral information, high working distance, and micron-scale resolution. We establish preliminary findings suggesting high spatial microvariability in coral tissue fractal dimension, indicating usage of the Henyey-Greenstein phase function to be unsuitable for modeling of light scattering in some regions of corals. The rapid imaging speed and low phototoxicity of ISOCT could be used in future studies to monitor real-time responses to stressors or other stimuli. Furthermore, spectroscopic analysis of the ISOCT signal could be used to infer nanoscale changes in cellular structure, such as dysregulated nuclear chromatin condensation, or altered absorption profiles from changes in pigment expression and Symbiodiniaceae distribution. The inherent quantitative and spatially-resolved nature of ISOCT will also prove useful in future study of photonic transport within the whole holobiont, an effect that until now has only been studied separately in coral skeletons and tissues. Our study provides a new tool that allows for non-invasive high-resolution quantification of chlorophyll *a* pigment density in live corals.

## Supplementary information

Supplementary material

Supplementary video 1

Supplementary video 2

Supplementary video 3

## Data Availability

The datasets generated during and/or analysed during the current study are available from the corresponding author on reasonable request.
