# Author Correction: Measuring light scattering and absorption in corals with Inverse Spectroscopic Optical Coherence Tomography (ISOCT): a new tool for non-invasive monitoring

**DOI:** 10.1038/s41598-019-54379-5

**Published:** 2019-11-27

**Authors:** G. L. C. Spicer, A. Eid, D. Wangpraseurt, T. D. Swain, J. A. Winkelmann, J. Yi, M. Kühl, L. A. Marcelino, V. Backman

**Affiliations:** 1 Department of Chemical and Biological Engineering, Northwestern University, Evanston, IL USA; 2 Department of Biomedical Engineering, Northwestern University, Evanston, IL USA; 3 Department of Chemistry, University of Cambridge, Lensfield Road, UK; 4 Scripps Institution of Oceanography, University of California, San Diego, USA; 5 Department of Civil and Environmental Engineering, Northwestern University, Evanston, IL USA; 6 Integrative Research Center, Field Museum of Natural History, Chicago, IL USA; 7 Department of Medicine, Boston University School of Medicine, Boston, MA USA; 8 Marine Biological Section, Department of Biology, University of Copenhagen, Helsingør, Denmark; 9 Climate Change Cluster, University of Technology Sydney, Ultimo, NSW Australia; 10 Wellman Center for Photomedicine, Harvard Medical School and Massachusetts General Hospital, Boston, MA USA; 11 Department of Marine and Environmental Sciences, Nova Southeastern University, Dania Beach, FL USA

Correction to: *Scientific Reports* 10.1038/s41598-019-50658-3, published online 02 October 2019

In the original version of this Article, Luisa Marcelino was incorrectly affiliated with ‘Department of Chemistry, University of Cambridge, Lensfield Road, UK’ and ‘Scripps Institution of Oceanography, University of California, San Diego, USA’. The correct affiliations are listed below.

Department of Civil and Environmental Engineering, Northwestern University, Evanston, IL, USA

Integrative Research Center, Field Museum of Natural History, Chicago, IL, USA

This error has now been corrected in the PDF and HTML versions of the Article.

