# Supplementary material for: Measuring light scattering and absorption in corals with Inverse Spectroscopic Optical Coherence Tomography (ISOCT): a new tool for non-invasive monitoring
Source: Sci Rep. 2019 Oct 2;9:14148. doi: 10.1038/s41598-019-50658-3 (PMC6775107; doi:10.1038/s41598-019-50658-3)
Supplement: Supplementary file 1 — Supplementary material [file 41598_2019_50658_MOESM1_ESM.pdf]

Supplementary Material

Measuring light scattering and absorption in corals with Inverse Spectroscopic Optical Coherence Tomography (ISOCT): a new tool for non-invasive monitoring

G.L.C. Spicer, A. Eid, D. Wangpraseurt, T. D. Swain, J. A. Winkelmann, J. Yi, M. Kühl, L. A. Marcelino*, and V. Backman*

*** Correspondence:** Corresponding Authors: l-marcelino@northwestern.edu, v-backman@northwestern.edu

1 Supplementary Methods

OCT system resolution (Supplementary figure 1)

To determine the lateral spatial resolution of the OCT system shown in supplementary figure 1(A), a razor edge was scanned at the objective focal plane with high sampling density (512 A lines collected in a 100 µm scan length) to create a resolution-limited OCT image of the razor edge intensity step function. OCT intensity along the axial dimension was summed over a 50 µm depth range to isolate the razor reflection signal from background noise, creating an *en face* projection image. This 2D *en face* image was summed along the edge dimension to average the edge step function for analysis. A Savitzky-Golay filter was applied to this edge response function to gently smooth the signal and calculate the first spatial derivative of intensity as a function of distance. The width of this first derivative peak characterizes the sharpness of the edge response function, quantifying lateral resolution of the OCT system assuming a sharp cutoff of true sample, high enough sampling density relative to system resolution, and an acceptably small filter kernel relative to resolution. The full width at half-maximum (FWHM) of the first spatial derivative peak is given as the lateral resolution of the OCT system.

Axial resolution of the OCT system shown in supplementary figure 1(B) was measured by scanning a mirror placed at the objective focal point with a neutral-density (ND) filter placed in the sample arm to attenuate signal, avoiding spectrometer detector saturation. The peak corresponding to mirror signal in a single OCT A-line signal was analyzed, the FWHM of which characterized the axial resolution of the system. All analysis was performed using Matlab software.

Validation of μ_t measurement (Supplementary figure 2)

Validation of μ_t measurement technique was performed by scanning aqueous suspensions of 200 µm diameter polystyrene latex beads with concentrations ranging from 0.1-1.0 wt%. A 50 µL droplet of the suspension of interest was pipetted onto a quartz plate and a 2 x 2 mm area was scanned with 256 x 256 pixels. The surface of the droplet was angled slightly and manual lateral segmentation of the *en face* image was later performed to ensure only clean signal that was free from specular reflection was included in μ_t analysis. The same code applied to coral image processing was used to calculate mean μ_t from beads samples. For each concentration the mean μ_t from 3 sample regions were averaged and standard error on the mean (SEM) was plotted as error bars in supplementary figure 2. Theoretical μ_t values for each concentration were calculated from Mie Theory and used as the ordinate axis for parity comparison.

Property maps from coral skeletons (Supplementary figure 3)

For projection maps of properties from coral skeletons shown in supplementary figure 3, the same analysis code described in materials and methods was applied to manually segmented lateral regions where the skeleton was visible in OCT B-scans. The surface of the skeleton underlying the live tissue layer was also manually segmented in depth to ensure accuracy of the skeletal surface location. The identity of the skeleton in these regions was confirmed by later removing the coral tissue by water jet while keeping the OCT scan of the same location.

Analysis of microvariability of *D* (Supplementary figure 5)

To confirm the true spatial variability of *D* values in the tissue of living coral, we performed an averaging box analysis on two-dimensional *D* maps of *Merulina ampliata* tissue and an aqueous suspension of 80 nm beads (2 wt%). For this analysis, a square box with given edge length was placed randomly within the 2D *D* map and an average *D* was calculated from all pixels contained within. This random sampling was repeated 1000 times for each edge length tested, and the standard deviation over these 1000 samples was interpreted as the square root of the local spatial variance in *D*. For the sample of 80 nm polystyrene beads, the expected number of beads contained within a resolution voxel of the OCT system is on the order of 10^4 so this medium is considered to be spatially homogeneous with constant *D*. Therefore, the spatial variance in *D* due to instrument noise and subsequent propagation through processing is fully characterized by this measurement of 80 nm beads shown in supplementary figure 5(C). The higher spatial variance in *D* seen from the same analysis applied to the *Merulina ampliata D* map is due to an additional spatial variance in sample structure quantified by *D*.

2 Supplementary Figures

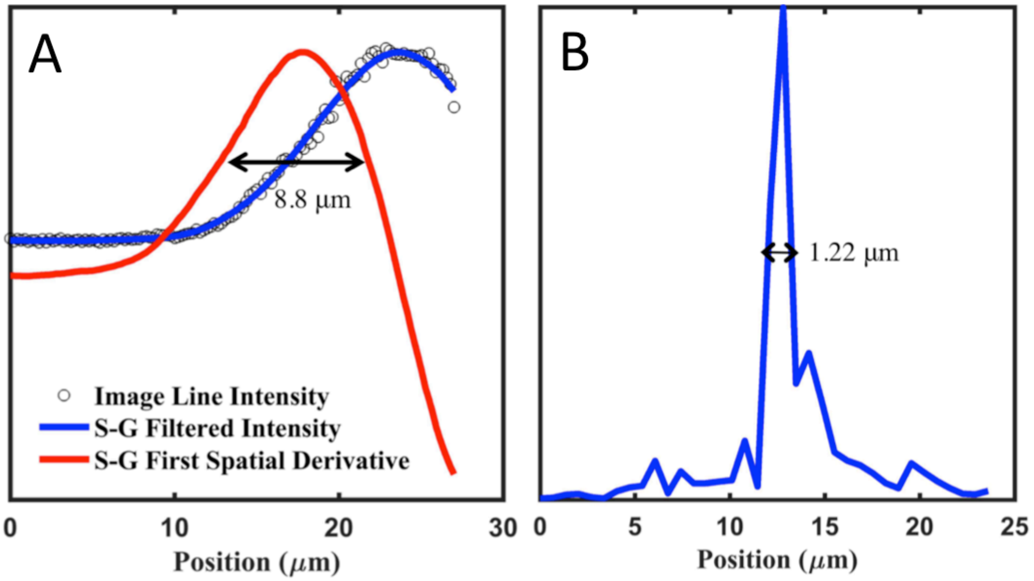

Supplementary figure 1. Quantification of spatial resolution of visible OCT system used in lateral (A) and axial (B) directions.

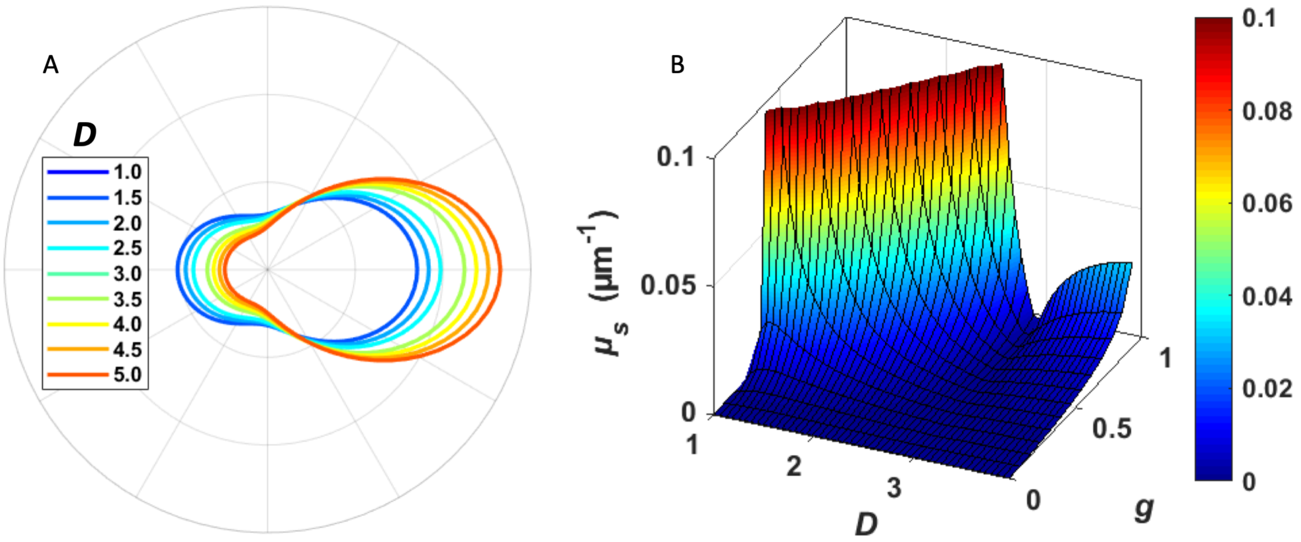

Supplementary figure 2. (A) Shape of scattering phase function, $p(\theta)$, for D values ranging from 1-5. Simulated phase function incorporates correlation length l_c of 50 nm and wavelength of 600 nm in the absence of absorption. (B) Surface plot shows behavior of scattering coefficient with respect to changes in D and g in the Born approximation as characterized theoretically in a previously reported derivation (Rogers et al. Opt. Lett. **34**(12), (2009)).

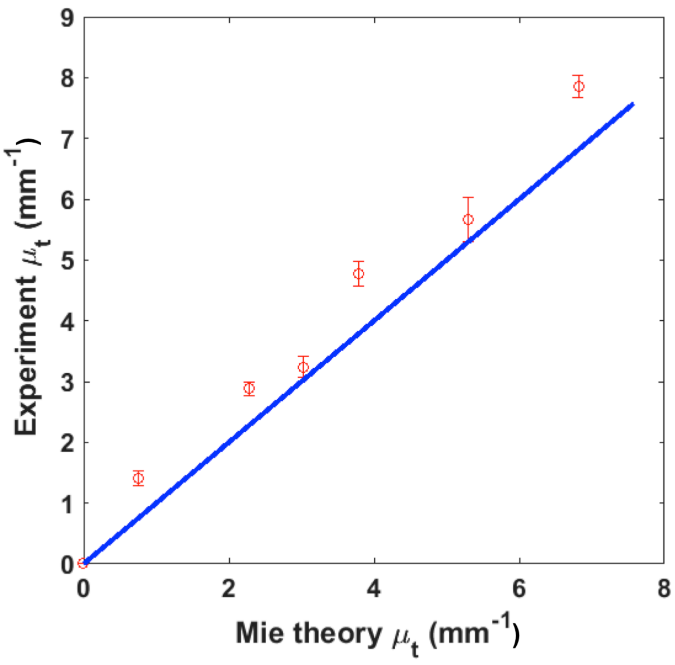

Supplementary figure 3. Validation of total attenuation coefficient measurement by comparison with Mie theory for samples of 200 nm polystyrene spheres of varying concentrations (0.1-1.0 wt%).

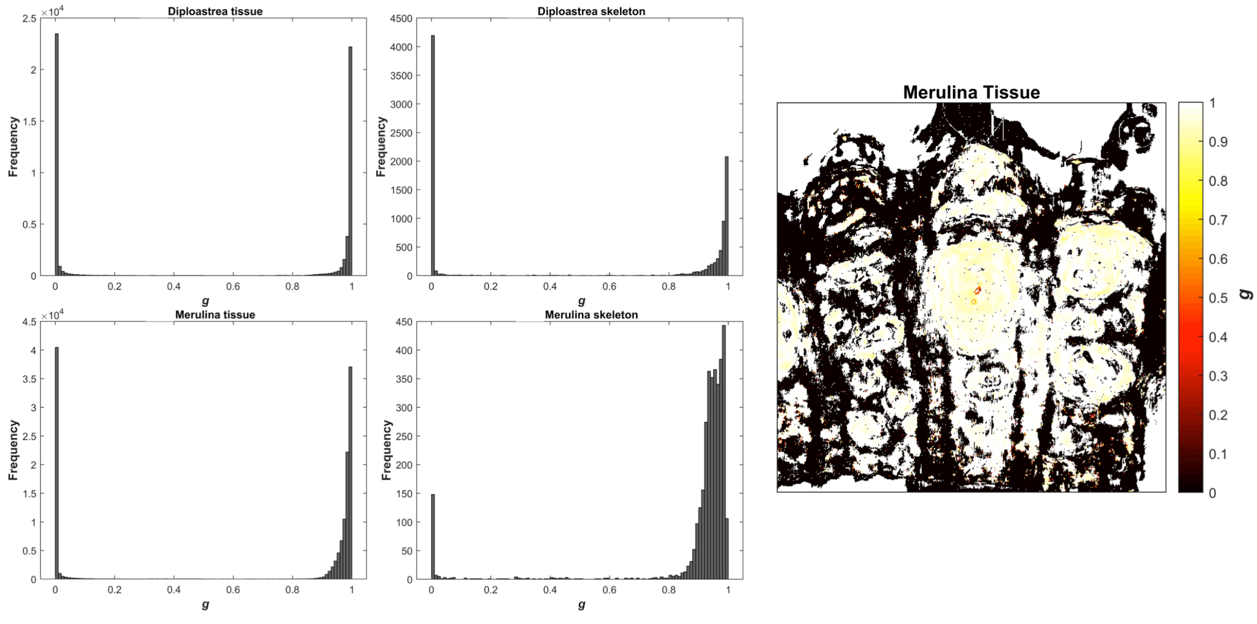

Supplementary figure 4. Histograms of scattering anisotropy g from tissue and skeleton of *Merulina ampliata* and *Diploastrea heliopora*. Projection map of g from tissue layer of *Merulina ampliata* (right).

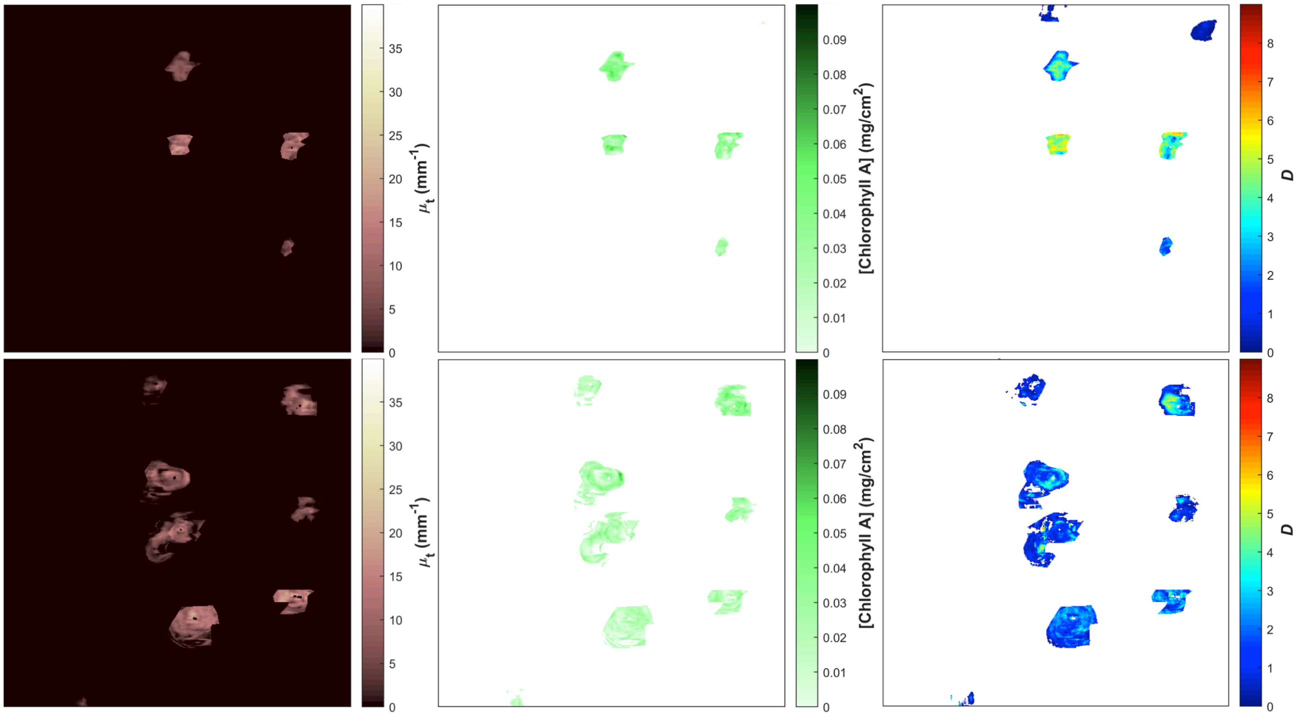

Supplementary figure 5. *En face* projection maps of μ_t, Chlorophyll A density, and *D* for visible skeletal ridges from *Merulina ampliata* (top row) and *Diploastrea heliopora* (bottom row).

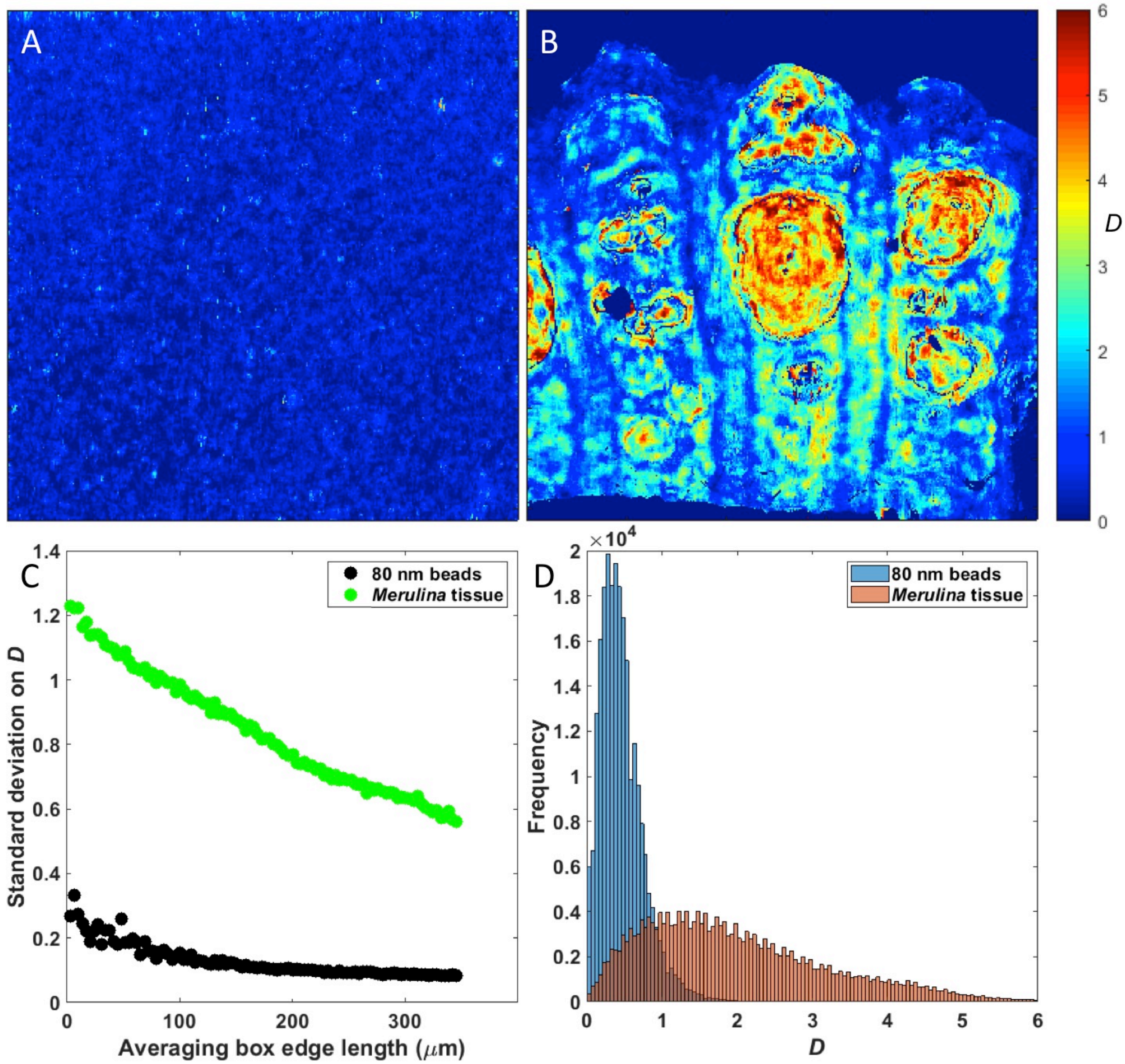

Supplementary figure 6. 2D maps of D from (A) 80 nm polystyrene beads suspension and (B) *Merulina ampliata* tissue. (C) Spatial variance comparison between *D* value maps from a homogenous sample of 80 nm polystyrene beads and *Merulina ampliata* tissue. (D) Histogram of *D* values from maps in (A) and (B).

Supplementary video 1. Flythrough of OCT B-scans collected from a live *Diploastrea heliopora* polyp mouth. B-scan image shown on left, *en face* projection with B-scan slice location demarcated with line shown on right.

Supplementary video 2. 3-D rendering of ISOCT scan of *Merulina ampliata* coenosarc. Traditional OCT image shown in grayscale, with color overlay corresponding to local *D* value.

Supplementary video 3. Coral skeleton identified by tissue removal. A side-by-side video of a flythrough of corresponding OCT B-scans showing the intact coral (left side) and stripped skeleton (right side).
